# *Channa Striatus* Protects Against PTZ-Induced Seizures in LPS Pre-conditioned Zebrafish Model

**DOI:** 10.3389/fphar.2022.821618

**Published:** 2022-04-04

**Authors:** Vanessa Lin Lin Lee, Anwar Norazit, Suzita Mohd Noor, Mohd. Farooq Shaikh

**Affiliations:** ^1^ Neuropharmacology Research Laboratory, Jeffrey Cheah School of Medicine and Health Sciences, Monash University Malaysia, Selangor, Malaysia; ^2^ Department of Biomedical Science, Faculty of Medicine, University of Malaya, Kuala Lumpur, Malaysia

**Keywords:** neuroinflammation, epilepsy, *Channa* striatus, seizures, anti-inflammatory, pentylenetetrazol

## Abstract

Epilepsy is a neurological disorder characterized by recurrent unprovoked seizures. Mounting evidence suggests the link between epileptogenesis and neuroinflammation. We hypothesize that eliminating neuroinflammation can alleviate seizure severity and prolong seizure onset. *Channa striatus* (CS) is a snakehead murrel commonly consumed by locals in Malaysia, believed to promote wound healing and mitigate inflammation. This study aims to unravel the anticonvulsive potential of CS extract on neuroinflammation-induced seizures using an adult zebrafish model. Neuroinflammation was induced *via* cerebroventricular microinjection of lipopolysaccharides from *E. coli* and later challenged with a second-hit pentylenetetrazol at a subconvulsive dose of 80 mg/kg. Zebrafish behaviour and swimming pattern analysis, as well as gene expression analysis, were done to study the pharmacological property of CS. CS extract pre-treatment in all doses significantly reduced seizure score, prolonged seizure onset time and slightly improved the locomotor swimming pattern of the zebrafish. CS extract pre-treatment at all doses significantly reduced the expression of NF_K_B gene in the brain, and CS extract at 25 mg/L significantly reduced the IL-1 gene expression suggesting anti-neuroinflammatory properties. However, there were no significant changes in the TNFα. Besides, CS extract at 50 mg/L also elevated the expression of the CREB gene, which exerts neuroprotective effects on the neurons and the NPY gene, which plays a role in modulating the inhibition of the excitatory neurotransmission. To sum up, CS extract demonstrated some anticonvulsive and anti-inflammatory activity on neuroinflammation-induced seizures. Still, more studies need to be done to elucidate the mechanism of action of CS extract.

## Introduction

Epilepsy is a dynamic brain disorder that affects approximately 50 million people worldwide (WHO, 2018). The main hallmark of epilepsy is the spontaneous, unprovoked and synchronic hyperactivity of the neurons caused by sudden and excessive electrical discharges (Yaksi et al., 2021). The root cause of epilepsy is idiopathic, but there is compelling evidence suggesting the link between epileptogenesis and a wide array of factors stemming from neuroinflammation (Lee and Shaikh, 2019). Over the past decade, the connection between inflammation and epilepsy has been extensively studied using *in vitro* and *in vivo* experimental models, as inflammation was said to be detrimental to cell survival, but at the same time, neuroinflammation was also believed to be neuroprotective (Vezzani et al., 2011).

The gold standard of treatment for epilepsy is anti-seizure drugs (ASDs). However, studies have shown that about one-third of individuals with epilepsy still experience seizures resistant to medication (Laxer et al., 2014). This is because they only provide symptomatic relief by blocking seizures rather than treating the underlying pathology of seizures (Pitkänen and Sutula, 2002). Hence, there is an urgent need to develop new therapies to alleviate the burden of drug-resistant epilepsy. In neuroinflammation-mediated epilepsy, it is believed that the inflammation promotes the release of cytokines and prostaglandins, which can lead to neuronal hyperexcitability and generation of seizures (Vezzani et al., 2011). Therefore, targeting and ameliorating neuroinflammation can eliminate the root cause of seizures and, at the same time, possibly treat neuroinflammation-mediated epilepsy.

In Malaysia, *Channa striatus* (CS), more commonly known as “haruan” fish by the locals, is an indigenous, predatory freshwater snakehead fish traditionally consumed for its pharmacological benefits. Numerous scientific studies have deciphered the therapeutic benefits of CS, such as its wound healing and analgesic ability, as well as boosting energy for the sick. In the lab, extracts of the fish can be made from the whole fish, roe, mucus and skin of the fish, but the most common preparation method is from the fish fillet, to mimic the traditional preparation method of making fish soup using the fillet (Mat Jais et al., 1997). Amino acids and fatty acids, found in high concentrations in the fish extract, might have contributed to its pharmacological properties. Important amino acids of the fish include glycine, alanine, lysine, aspartic acid and proline. In contrast, its major fatty acids are palmitic acid, oleic acid, stearic acid, linoleic acid and arachidonic acid (Zakaria et al., 2007). These amino acids are the essential component of collagen found in human skin. For example, glycine and other amino acids combine to form a polypeptide associated and responsible for skin growth and wound healing (Chyun and Griminger, 1984; Zakaria et al., 2007). The fatty acid, arachidonic acid, is a precursor to prostaglandins which may induce platelet aggregation and adhesion to endothelial tissue to initiate blood clotting (Zakaria et al., 2007). Although the prostaglandins derived from the breakdown of arachidonic acid are said to be mediators of nociception and inflammation, numerous studies are showing the antinociceptive, anti-inflammatory and wound healing effect of Haruan extract (HE) (Mat Jais et al., 1997; Baie and Sheikh, 2000; Baie and Sheikh, 2000; Somchit et al., 2004; Zakaria et al., 2004; Zakaria et al., 2005).

In this study, adult zebrafish (*Danio rerio*) was used as an animal model. Zebrafish have 70% genes homologous to humans, with approximately 85% genes related to recognized epilepsy genes (Hortopan et al., 2010; Howe et al., 2013). To unravel the anticonvulsive potential of CS extract under inflammatory conditions, we established a neuroinflammation model using adult zebrafish, challenged with a second-hit pentylenetetrazol (PTZ) at a subconvulsive dose.

## Materials and Methods

### Chemicals and Equipment

Professor Abdull Manan Mat Jais from Abmanan Biomedical Sdn. Bhd. (Malaysia), provided CS extract which was extracted following a protocol described previously (Mat Jais et al., 1997). Pentylenetetrazole (PTZ) was used as a proconvulsant and purchased from Sigma-Aldrich (St. Louis, MO, United States ) and lipopolysaccharide (LPS) derived from *Escherichia coli* 026: B6 was obtained from Sigma Aldrich (St. Louis, United States ). Levetiracetam (Lev; Keppra^®^) was used as a positive control and manufactured by UCB Pharma (Brussels, Belgium). The zebrafish behavior was recorded using Sony Digital Handycam video camera (HDR-PJ340E) and later analyzed using SMART V3.0.05 tracking software (Panlab, Harvard Apparatus, Massachusetts, United States). For the cerebroventricular microinjection, stereoscopic microscope Nikon, SMZ 1500 (Nikon, Tokyo, Japan), pneumatic microjector, IM-11-2 (Narishinge, Tokyo, Japan) and borosilicate capillaries G-1 (Narishinge, Tokyo, Japan) were used.

### Animals

Heterogeneous wild-type adult zebrafish (*Danio rerio*) of approximately 8 months old were purchased from *Danio* Assay (UPM, Malaysia). The zebrafish were housed at the animal facility unit of Monash University Malaysia under standardized husbandry conditions. Fish tanks (36 cm × 26 cm x 22 cm) were used to hold the zebrafish. The water temperature was maintained between 26 and 28°C, and pH 6.8 and 7.1 with a 14:10 light to dark cycle. They were fed three times a day with TetraMin^®^ Tropical Flakes with occasional supplementation of live brine shrimps (artemia). Constant aeration was provided to the tanks with water circulation and filtration system. All animal experimentations were approved by the Monash University Malaysia Animal Ethics Committee (Project ID: 18499). Before any invasive procedures, the zebrafish were anaesthetized with 0.6 mg/L of benzocaine and precautions were taken to minimise suffering.

### Neuroinflammation Induction With Cerebroventricular Microinjection (CVMI)

Adapted from Kizil et al., 2013 study, cerebroventricular microinjection was performed on the adult zebrafish to induce neuroinflammation. Lipopolysaccharide (LPS) derived from *Escherichia coli* 026: B6 (Sigma Aldrich, St. Louis, United States ) was used as an inflammatory agent for the induction. Firstly, a small opening was incised on the zebrafish skull using a 30G needle on the cranial bone close to the midline located above the optic tectum. The slit exposes the zebrafish’s cerebroventricular fluid (CVF), allowing LPS solution to be microinjected using thin glass capillaries without damaging the brain. This injection can disperse the LPS solution throughout the brain, targeting the ventricular and periventricular cells, inducing generalized neuroinflammation. For this study, 100 nL of 2 mg/ml LPS were injected into zebrafish in groups 6-10, and distilled water of the same volume was injected into zebrafish in group 5.

### Anti-Convulsant Study

#### Drug Treatment and Groups

The zebrafish were divided into ten groups, consisting of 8 zebrafish per group. Pentylenetetrazol (PTZ, Sigma Aldrich, United States ) was used as a chemiconvulsant to induce seizures. PTZ was dissolved in distilled water to a concentration of 80 mg/kg and injected via the intraperitoneal route. This study used Levetiracetam (Lev, Keppra^®^) from UCB Pharma (Braine-l’Alleud, Belgium) as a positive control. It was administered by dissolving in system water (5 g/L) and poured into a tank where the zebrafish were placed. CS extract treatment was also given by dissolving into system water and poured into the zebrafish tank. The zebrafish were exposed to CS extract for 2 h.

Group 1: ControlGroup 2: LPS onlyGroup 3: Lev onlyGroup 4: CS (100 mg/ml) onlyGroup 5: Sham + PTZ (80 mg/kg)Group 6: LPS + PTZ (80 mg/kg)Group 7: LPS + Lev + PTZ (80 mg/kg)Group 8: LPS + CS 25 mg/ml + PTZ (80 mg/kg)Group 9: LPS + CS 50 mg/ml + PTZ (80 mg/kg)Group 10: LPS + CS 100 mg/ml + PTZ (80 mg/kg).

The anti-convulsant study was adapted from the protocol established in our lab (Chung et al., 2020). Group 1 acted as the control group and did not receive any treatments. Groups 2, 3 and 4 acted as the per se group and received only one treatment: LPS, lev, and CS extract, respectively. Group 5 was the sham control, receiving saline during the CVMI. Group 6 and 7 were negative and positive controls, respectively. Groups 8, 9 and 10 were treatment groups receiving three different doses of CS extract representing low, medium and high doses. LPS administration via CVMI was given 24 h before treatments to allow the development of neuroinflammation. Lev and CS extract treatment lasted for 2 h before the PTZ administration.

In this study, PTZ was administered at 80 mg/kg ip., a subconvulsive dose. Usually, a subvconvulsive dose is given to induce chronic seizures, but in our study, the subconvulsive dose allowed us to investigate if the neuroinflammation aggravated the susceptibility to seizures. After administering seizures, the zebrafish movements were recorded using a Sony Digital Handycam video camera (HDR-PJ340E) for behavioural analysis. Then, the zebrafish brains were harvested for gene expression analysis.

### Seizure Behavioural Analysis

The following scoring system was used to evaluate the zebrafish seizure scores, which indicated the severity of seizures:

Score 1—Short swim mainly at the bottom of the tankScore 2—Elevated swimming activity and increased frequency of opercular movements Score 3—Burst swimming patterns with left and right and erratic movements Score 4—Circular swimming movements.

The highest seizure score displayed and the onset time for seizure score 4 (if any) were noted when viewing the recorded videos. Then, the videos were analyzed using tracking software SMART V3.0.05 (Pan Lab, Harvard Apparatus) to evaluate the swimming patterns of the zebrafish and the total distance travelled.

### Gene Expression Analysis

#### Brain Harvesting

The extracted individual fish brains were immediately transferred to a 200 μl of ice-cold TRIzol^®^ (Invitrogen, United States ) for gene expression analysis and stored at -80 °C until further investigation.

#### RNA Isolation and Synthesis of the First-Strand cDNA

According to the protocol supplied by the kit’s manufacturer (Qiagen, United States ), the mRNA was isolated and was identical to the protocol used by Kundap et al. (2017). Briefly, the zebrafish brain was first homogenized in TRIzol^®^ before the isolation of mRNA. The mRNA obtained was quantified with Nanodrop Spectrophotometer was then converted to cDNA as per the instructions given in the Omniscript Reverse-transcription Kit (Qiagen, United States ).

#### StepOne^®^ Real-Time PCR

The gene expression levels were determined via real-time quantitative RT-PCR (Applied Biosystems, United States ). The QuantiNova SYBR^®^ Green PCR Kit and the appropriate Qiagen primer set were used for each gene; using a similar protocol as Choo et al. (2018). The genes that were studied are nuclear factor kappa B (NF-κB), tumour necrosis factor-alpha (TNFα), interleukin (IL-1), high mobility group box 1 (HMBG1), neuropeptide Y (NPY), and cAMP Response Element-Binding Protein (CREB). Eukaryotic translation elongation factor 1 alpha 1b (eef1a1b) was used as the housekeeping gene. The following are their respective primers used for the PCR:

NF-κB: Dr_nfkb1_2_SG QuantiTect Primer Assay (Cat no. QT02498762).
TNF-α: Dr_tnf_1_SG QuantiTect Primer Assay (Cat no. QT02097655).
IL-1: Dr_il1rapl1a_1_SG QuantiTect Primer Assay (Cat no. QT02131850).
HMGB1: Dr_hmgb1b_2_SG QuantiTect Primer Assay (Cat no: QT02088555).
NPY: Dr_npy_1_SG QuantiTect Primer Assay (Cat no. QT02205763).
CREB_1: Dr_crebbpa_1_SG QuantiTect Primer Assay (Cat no. QT02197503).
eef1a1b: Dr_eef1a1b_2_SG QuantiTect Primer Assay (Cat no. QT02042684).

The PCR began with first incubation at 95°C for 2 min before thermal cycling. According to the manufacturer’s protocol, the thermal cycling settings for the PCR were 40 cycles of 95°C for 5 s and 60°C for 15 s. We calculated the relative expression level (fold change) of the genes of interest by normalizing the threshold cycle (Ct) values obtained from the genes of interest against the Ct value of the eef1a1b housekeeping gene. The formula for the calculation is:

Relative expression level=2^(Ct eef1a1b−Ct Gene of interest)

## Results

### Anti-Convulsant Study

**FIGURE 1:**
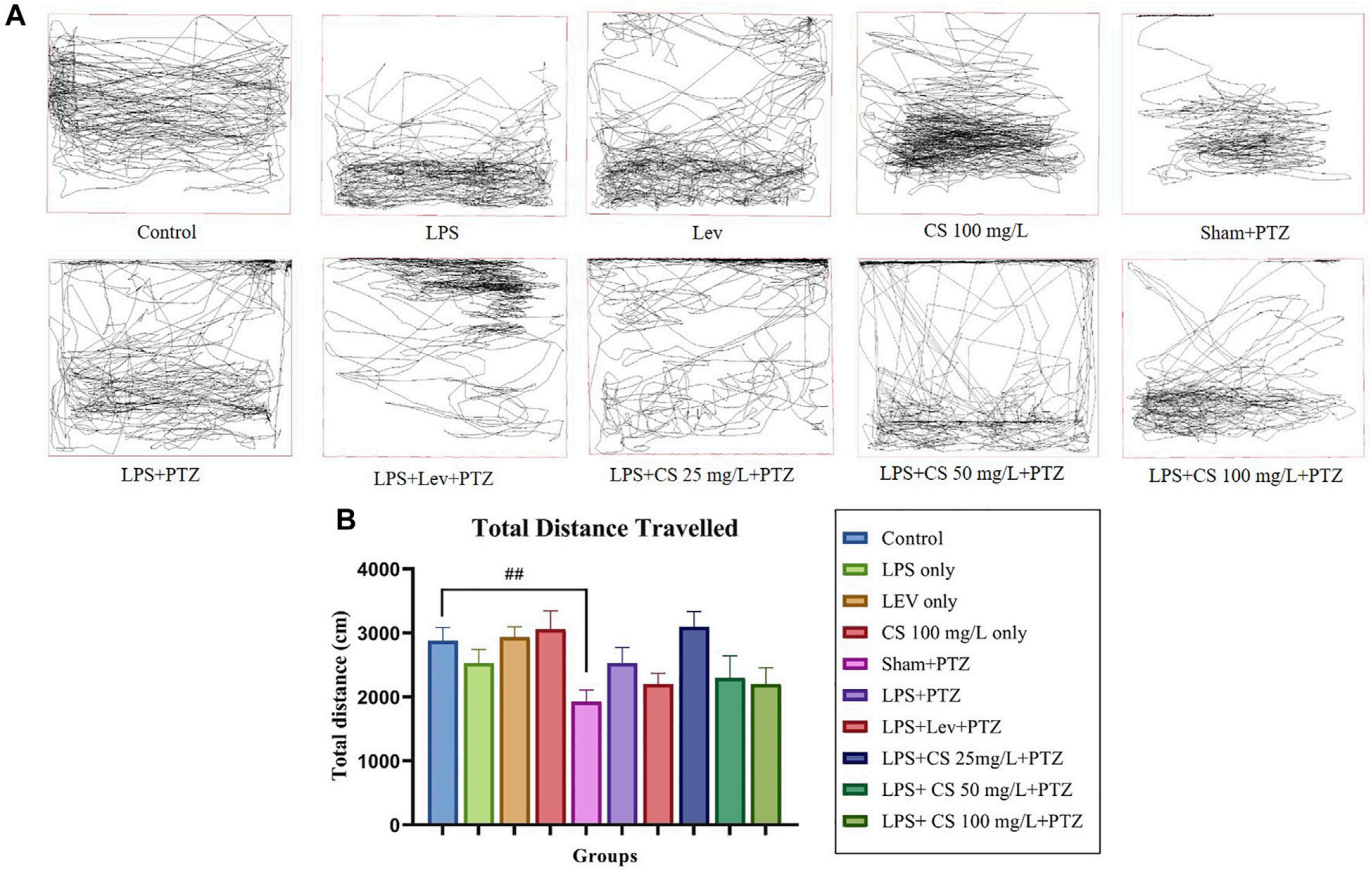
**(A)** Representative swimming pattern of zebrafish in each group. **(B)** Mean total distance travelled (cm) by zebrafish in each group. The *p*-values ^****^ *p* < 0.0001, ^***^ *p* < 0.001, ^**^ *p* < 0.01, and ^*^ *p* < 0.05 were regarded as statistically significant for the treatment groups compared to the negative control (LPS + PTZ). The *p*-values ^####^ *p* < 0.0001, ^###^ *p* < 0.001, ^##^ *p* < 0.01 and ^#^ *p* < 0.05 was regarded as statistically significant for the *per se* groups compared to the control.

Zebrafish swimming pattern was analyzed by SMART software, and one swimming pattern representative was selected for each group to be displayed in Figure 1. Zebrafish in the control group showed no preference toward any part of the tank. This observation can similarly be seen in zebrafish in lev, CS 100 mg/L and sham + PTZ groups. Conversely, zebrafish in the LPS group showed a preference for the lower part of the tank. Zebrafish from the negative control (LPS + PTZ) group showed a more erratic swimming pattern with more dwelling activity on the top of the tank. Lev pre-treatment reduced swimming activity, but top dwelling was still observed. Zebrafish in the CS extract pre-treated groups showed less erratic swimming behaviour, but top dwelling was still observed in groups treated with 25 mg/L and 50 mg/L. Zebrafish pre-treated with 100 mg/L did not show top dwelling activity. The mean total distance travelled was analyzed and compared. There was a significant reduction in the mean total distance travelled in the sham + PTZ group (*p* < 0.01). The changes in mean total distance travelled were insignificant in other groups.

**FIGURE 2:**
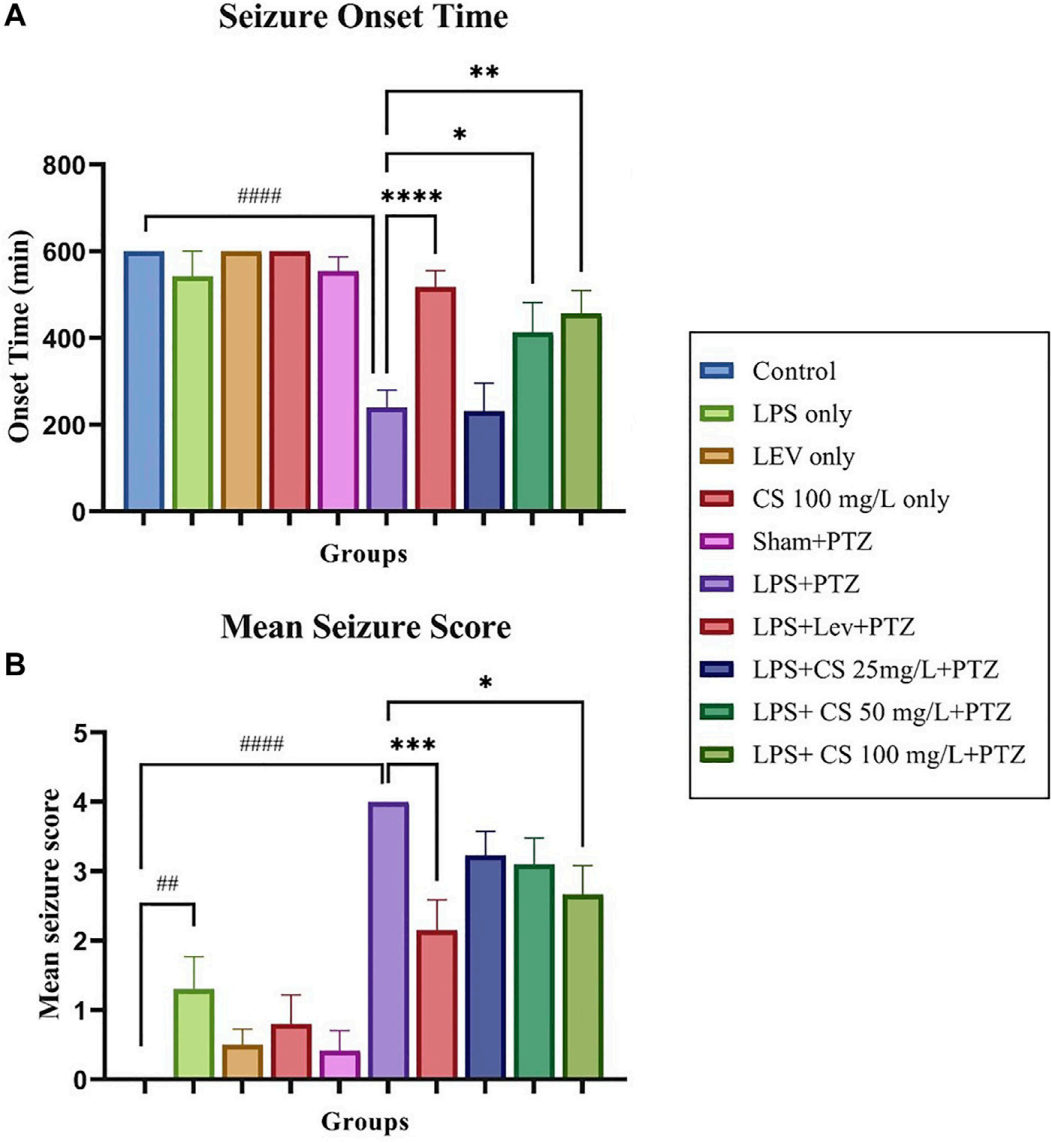
**(A)** Seizure score 4 onset time for each group. **(B)** Mean seizure scores for each group. The *p*-values ^****^ *p* < 0.0001, ***p < 0.001, ^**^ *p* < 0.01, and ^*^ *p* < 0.05 were regarded as statistically significant for the treatment groups compared to the negative control (LPS + PTZ). The *p*-values ^####^ *p* < 0.0001, ^###^ *p* < 0.001, ^##^ *p* < 0.01 and ^#^ *p* < 0.05 was regarded as statistically significant for the *per se* groups compared to the control.

The swimming behaviour of the zebrafish was observed for 10 min post recovery from anaesthesia as the acute seizures induced via PTZ usually last less than that. Therefore, zebrafish with no seizures were reported to have an onset time of 600 s. Our study observed no significant changes in the seizure onset time between the control and groups receiving only LPS, lev or CS extract treatments as shown in Figure 2. There is a significant dip in seizure onset time for the negative control (LPS + PTZ) group compared to the control (*p* < 0.0001). Levetiracetam treatment post-CVMI was able to delay the seizure onset time significantly (*p* < 0.001), and this is also observed in zebrafish receiving CS extract treatment of 50 mg/ml (*p* < 0.05) and 100 mg/ml (*p* < 0.01). As for mean seizure score, CVMI of LPS and PTZ administration induced seizure score 4 significantly (*p* < 0.0001) even at a subconvulsive dose of 80 mg/kg. Levetiracetam treatment reduced the mean seizure score significantly (*p* < 0.001). This pattern is also observed in CS extract treatment at all doses, with a significance of *p* < 0.05 at the dose of 100 mg/ml.

### Gene Expression Analysis

**FIGURE 3:**
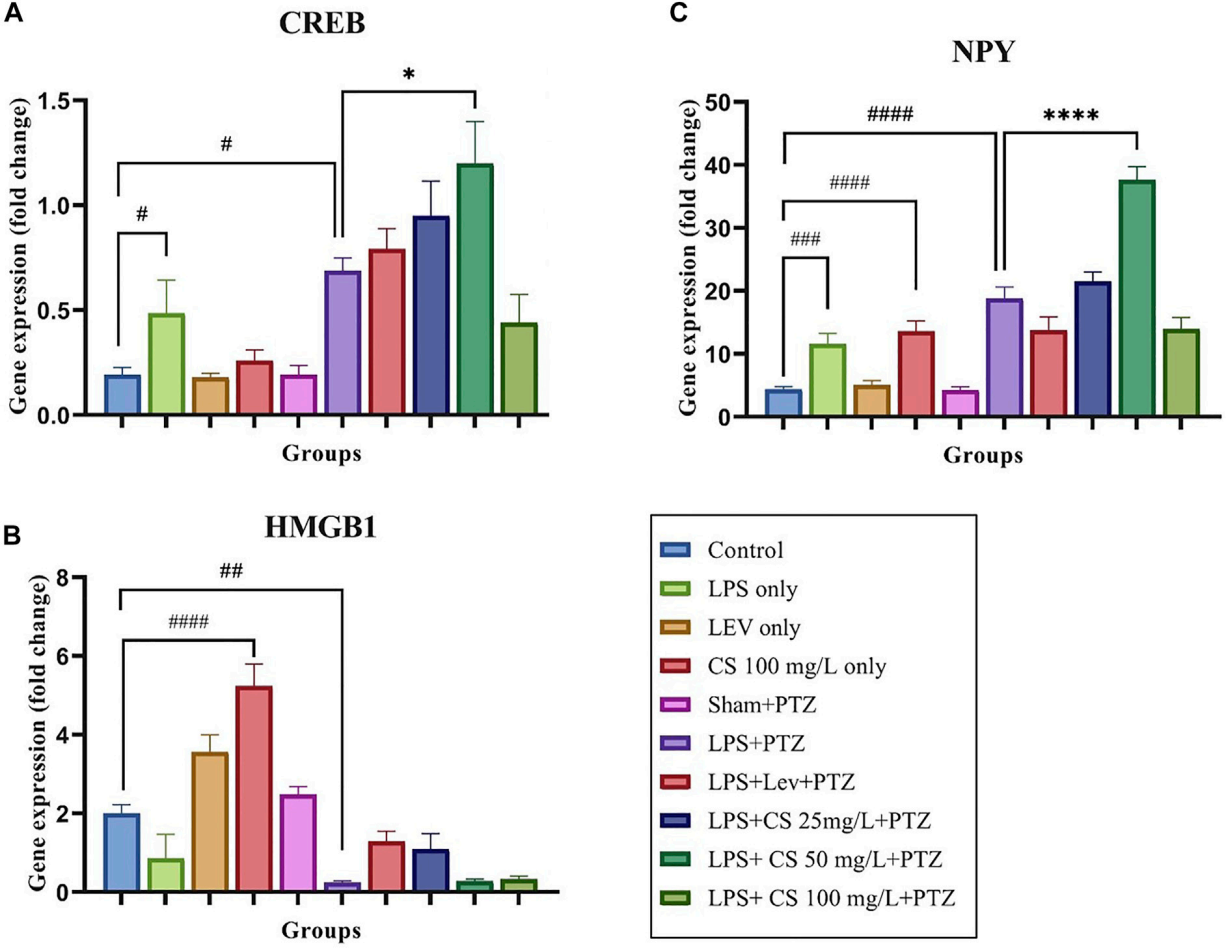
Gene expression levels for the following genes: **(A)** CREB, **(B)** NPY, and **(C)** HMGB1. The *p*-values ^****^ *p* < 0.0001, ^***^ *p* < 0.001, ^**^ *p* < 0.01, and ^*^ *p* < 0.05 were regarded as statistically significant for the treatment groups compared to the negative control (LPS + PTZ). The *p*-values ^####^ *p* < 0.0001, ^###^ *p* < 0.001, ^##^ *p* < 0.01 and ^#^ *p* < 0.05 was regarded as statistically significant for the *per se* groups compared to the control.

A Gene expression study was performed to evaluate the changes in the expression levels of several inflammatory genes and genes implicated in epilepsy. As per Figure 3A, the expression of CREB was upregulated significantly in zebrafish in the negative control (LPS + PTZ) compared to the control group (*p* < 0.05). Interestingly, the zebrafish in the LPS only group had a significantly higher expression of CREB (*p* < 0.005). In contrast, other *per se* groups did not show any significant changes compared to the control group. The treatment of lev and CS extract further increased the expression of CREB, and this is significant at the CS extract dose of 50 mg/ml (*p* < 0.05) compared to the negative control (LPS + PTZ). CS extract treatment at 100 mg/ml reduces the CREB expression to a level lower than the negative control (LPS + PTZ), but this is not significant.

As per Figure 3B, the expression of HMGB1 was significantly upregulated in zebrafish receiving CS extract treatment at 100 mg/ml (*p* < 0.0001) compared to the control group. For the negative control (LPS + PTZ), we found a significant downregulation of the expression of HMGB1 compared to the control group (*p* < 0.01). Lev and CS extract at doses of 25 mg/ml treatments slightly increased the expression of HMGB1, but these increments were not significant compared to the negative control (LPS + PTZ).

As illustrated in Figure 3C, the expression of the NPY gene in the negative control (LPS + PTZ) group was significantly higher than in the control group (*p* < 0.0001). A similar pattern was observed in the LPS only and CS treatment at 100 mg/ml *per se*, compared to the control group. Lev treatment slightly reduced the expression of the NPY gene, while CS treatment at 50 mg/L significantly increased the NPY expression (*p* < 0.0001) with no significant changes at other concentrations.

**FIGURE 4:**
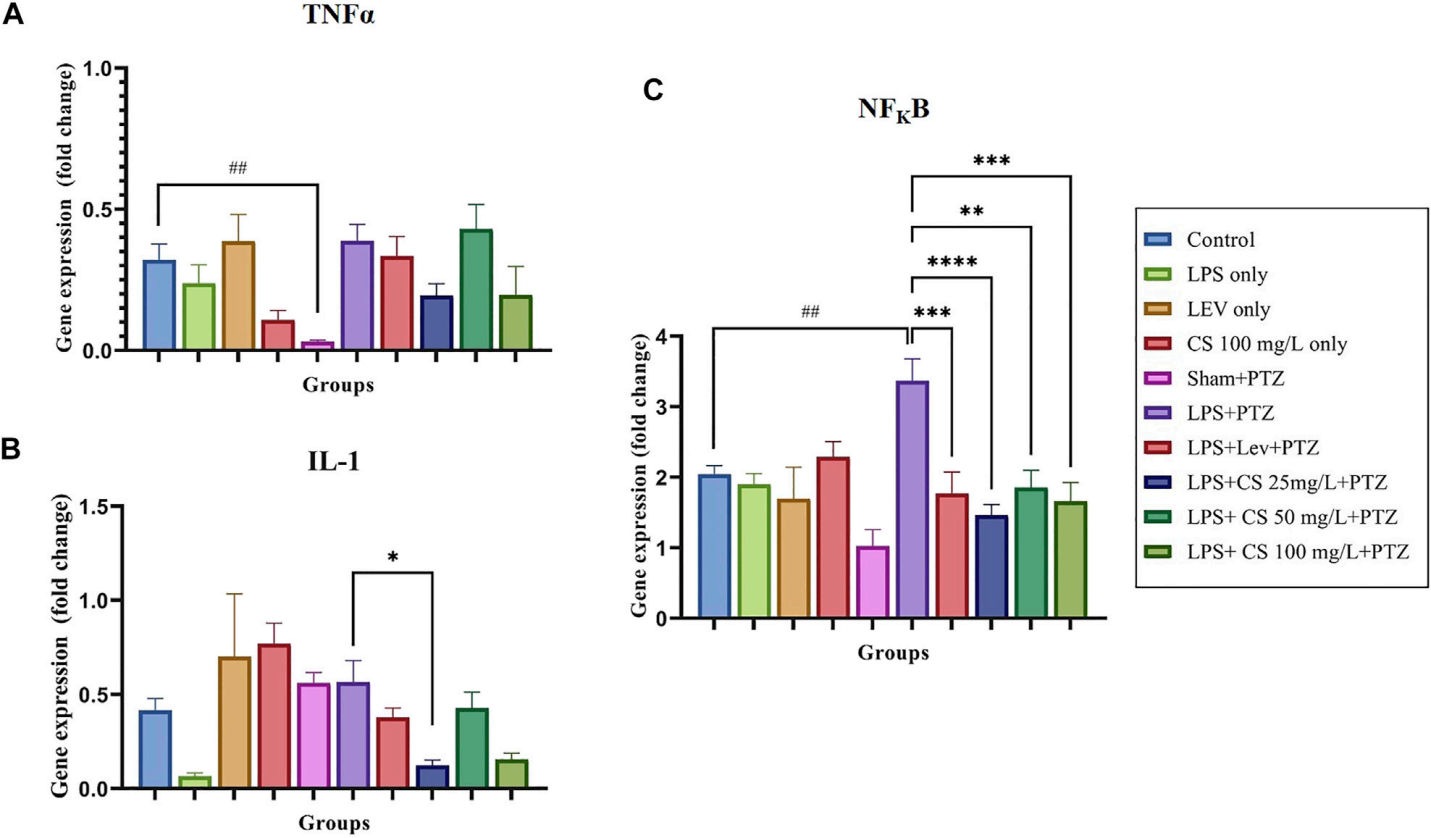
Gene expression levels for the following inflammatory genes: **(A)** TNFα, **(B)** NF_K_B, and **(C)** IL1. The *p*-values ^****^ *p* < 0.0001, ^***^ *p* < 0.001, ^**^ *p* < 0.01, and ^*^ *p* < 0.05 were regarded as statistically significant for the treatment groups compared to the negative control (LPS + PTZ). The *p*-values ^####^ *p* < 0.0001, ^###^ *p* < 0.001, ^##^ *p* < 0.01 and ^#^ *p* < 0.05 was regarded as statistically significant for the *per se* groups compared to the control.

The expression of inflammatory genes TNFα, IL-1 and NF_K_B, were demonstrated in Figures 4A–C, respectively. TNFα gene expression was significantly reduced in the Sham + PTZ group, compared to control (*p* < 0.01). In the negative control group, the TNFα expression was increased slightly and not significant compared to the control group. Lev and CS extract at a low dose of 25 mg/L and 100 mg/L reduced the TNFα expression but were not significant.

IL-1 expression was upregulated in the lev *per se* group, compared to the control (*p* < 0.01). There were no significant changes in the other *per se* groups. The expression of IL-1 was unexpectedly decreased in the negative control group (LPS + PTZ) compared to the control group, but this was not significant. There were no significant changes in the treatment groups except groups pre-treated with 50 mg/L of CS extract (*p* < 0.05).

For the expression of NF_K_B, we observed no significant changes in the *per se* groups compared to the control. The expression of NF_K_B was significantly elevated in the negative control (LPS + PTZ) group compared to the control (*p* < 0.01). Lev and CS extract treatments significantly lowered the expression of NF_K_B at a significance of *p* < 0.01 for CS extract 50 mg/L, *p* < 0.001 for lev and CS extract 100 mg/ml and *p* < 0.0001 for CS extract 25 mg/L.

## Discussion

This work aims to determine the anti-seizure potential of CS extract on seizures induced by PTZ aggravated by neuroinflammation. In our previous study (unpublished), we had established that 652.9 mg/L is the LD_50_ for CS extract in adult zebrafish, from which we selected 25 mg/L, 50 mg/L and 100 mg/L as the low, medium, and high dose for treatment in this study.

Firstly, neuroinflammation with the second hit PTZ model of adult zebrafish was established. We adapted the use of LPS, which is an inflammatory agent, and the CVMI procedure, to induce neuroinflammation to adult zebrafish. LPS can trigger the inflammation cascade of zebrafish embryos and inflict neuroinflammation in adult zebrafish via systemic injections (Ko et al., 2017; Fasolo et al., 2021). Considering PTZ will be injected intraperitoneally, we decided not to induce neuroinflammation systematically with LPS ip. But via CVMI instead, to prevent double injections to the same injection site and for targeted neuroinflammation induction (Kizil et al., 2013). After induced neuroinflammation, we challenge the zebrafish with a subconvulsive dose of PTZ (80 mg/kg). Based on the findings from the behavioural study, LPS CVMI alone does not produce clonic-like seizures (score 4) but relatively mild symptoms of seizures (Score 1 and 2). However, after a challenge with a second hit PTZ, clonic-like seizures were significantly induced. Therefore, this result suggested that the model for neuroinflammation-induced seizures is established. This is a novel model as no previously published evidence suggested such a model.

Swimming pattern analysis showed that zebrafish in the LPS *per se* group showed an increase in bottom-dwelling time. This is a classical characteristic of anxiety in zebrafish (Choo et al., 2019). The swimming pattern of zebrafish in the negative control (LPS + PTZ) group showed more erratic movements, and this can be explained by the inhibition of GABA by the action of PTZ, which promotes neuronal excitability leading to uncontrolled motor movements such as kindling or twitching (Chung et al., 2020). Lev pre-treatment seemed to alleviate the anxiety behaviour, which was also seen in groups pre-treated with CS extract at all doses, although the effects were mild. Coupled with the significant reduction of seizure scores and increase in seizure onset time, CS extract could exhibit anticonvulsant activity which were comparable to levetiracetam, an antiepileptic drug with anti-inflammatory property.

Based on the results of gene expression analysis, we observed a significant increase in the expression of CREB in the negative control group (LPS + PTZ) compared to the control group (*p* < 0.05). This was expected as CREB is believed to be involved in the epileptogenesis process (Guo et al., 2014). There was also a significant rise in CREB expression in the LPS *per se* group compared to the control (*p* < 0.05). This could be due to the neuroinflammation induced by LPS, which triggered the rise in CREB expression as it is a neuroprotective transcription factor (Feldmann et al., 2019). Lev and CS pre-treatment further increased the expression of the CREB gene, and this increment is significant at CS extract at a dose of 50 mg/L (*p* < 0.05). This finding establishes the neuroprotective potential of CS extract, which can similarly be seen in antiepileptic drugs such as cenobamate (Wiciński et al., 2021). NPY plays a role in modulating the different processes in the brain and is expressed in multiple areas of the brain. One of its major roles is regulating the inhibition of excitatory synaptic transmission, particularly glutaminergic synaptic transmission (Tekgul et al., 2020; Cattaneo et al., 2021). In the present study, NPY expression was found to be significantly increased in the negative control group (LPS + PTZ) compared to the control (*p* < 0.0001) and lev treatment was able to reduce the expression of NPY insignificantly. This finding was similar to that of a study by Tekgul et al. (2020). Interestingly, CS extract treatments further increased the expression of NPY at 25 mg/L (insignificant) and 50 mg/L (significant with *p* < 0.0001). Worsened seizure conditions usually accompany this observation, but adequate seizure control was observed with these groups. This could suggest that CS extract exerts its anticonvulsant activity via the enhanced activity of NPY and inhibitory processes.

HMGB1 is an endogenous protein that acts as a ‘danger signal’ in response to stress or damage to the neurons (Lee and Shaikh, 2019). Usually elevated in epileptic conditions, HMGB1 activates IL-1 and toll-like receptor 4 (TLR4) pathways and triggers the generation of seizures (Paudel et al., 2018). However, in our study, we found a significant reduction in HMGB1 expression in the negative control (LPS + PTZ) group compared to the control (*p* < 0.01). This is not uncalled for, as there were reports of a reduction of HMGB1 after kainic acid-induced seizures. A study by Luo et al. (2014) observed a drop in HMGB1 levels in the brain and a surge of serum HMGB1 levels. It was reported that HMGB1 was sequestered from neurons into the blood after acute cell death, explaining our results (Luo et al., 2014). The involvement of HMGB1 in the IL-1 pathway is noteworthy. The drop in brain levels of HMGB1 can explain the lack of a significant change in IL-1 of zebrafish in the negative control (LPS + PTZ) group compared to the control. Similar observations were also previously reported in a study by Choo et al. (2018). CS extract pre-treatment can reduce the expression of the inflammatory gene IL-1, and this is significant at 25 mg/L compared to the negative control (LPS + PTZ) group (*p* < 0.05). This can be due to the potent anti-inflammatory property of CS extract that was previously reported (Zakaria et al., 2008; Shafri and Abdul Manan, 2012).

The gene expression analysis also demonstrated an insignificant change in the inflammatory gene, TNFα, expression in the negative control group (LPS + PTZ) compared to the control. This was unexpected because acute seizures are most likely to increase the expression of TNFα. This may be contributed by the fact that the induction of neuroinflammation and second hit PTZ was insufficient to cause an increase in TNFα expression levels. On the other hand, we observed a significant increase in NF_K_B expression level in the negative control (LPS + PTZ) group compared to the control (*p* < 0.01). Lev and CS extract pre-treatment significantly reduced the NF_K_B expression level at significance of *p* < 0.001 for lev and CS extract 100 mg/L, *p* < 0.0001 for CS extract 25 mg/L, and *p* < 0.01 for CS extract 50 mg/L. Similar observations have been reported by Teocchi et al. (2013). This is most likely contributed by the anti-inflammatory and potential anticonvulsant properties of CS extract.

The present study demonstrated a novel model of neuroinflammation with second-hit PTZ in adult zebrafish. This model could be helpful in future studies involving neuroinflammation-induced epilepsy. While CS extract was shown to exhibit some anti-inflammatory and anti-convulsant properties, more studies need to confirm these findings. A protein expression study would be useful in studying the different proteins modulated by CS extract, revealing the pathways in which CS extract is involved.

## Conclusion

Overall, it may be said that CS extract can have an anti-neuroinflammatory and anticonvulsive effect as it was protective against seizures induced by the second-hit PTZ model. CS extract treatment displayed neuroprotective and anti-neuroinflammatory properties by modulating gene expression levels.

## Data Availability

The original contributions presented in the study are included in the article/Supplementary Material, further inquiries can be directed to the corresponding author.
